# Evaluation of urinary continence status and its influence on quality of life after gyneco-oncological treatment of female pelvic malignancies at an oncological center

**DOI:** 10.1186/s12905-022-01999-1

**Published:** 2022-10-25

**Authors:** Maryam Zafarnia, Lieven N. Kennes, Elmar Stickeler, Johannes Hoff, Laila Najjari

**Affiliations:** 1 Department of Gynecology and Obstetrics, Medical Faculty, RWTH Aachen University, Pauwelsstraße 30, 52074 Aachen, Germany; 2 Department of Economics and Business Administration, University of Applied Sciences Stralsund, Stralsund, Germany

**Keywords:** Urinary incontinence, Gyneco-oncological therapy, ICIQ-SF, Quality of life

## Abstract

Urinary incontinence (UI) could negatively affect a person’s quality of life (QoL). This study investigates the association among gynecological cancers, their treatments, UI, and its effect on the QoL of survivors of gynecological cancer. This cross-sectional questionnaire-based study included 121 cases from 405 patients who had undergone gyneco-oncological therapy. The participants were asked whether they experienced any form of UI and whether it impacted their QoL. The following therapies were used to treat the 12 gynecologic tumor types found in the participants: surgery (n = 116, 95.87%), chemotherapy (CTx) (n = 51, 42.2%), radiotherapy (RTx) (n = 31, 25.6%), and antibody therapy (ABT) (n = 11, 9.1%). No significant association was determined between tumor type and UI. However, body mass index (BMI), radical hysterectomy, vulvar or vaginal surgery, and presence of UI before treatment had significant impacts on the presence of UI. The surgical access routes, CTx, ABT, and hysterectomy had significant impacts on the severity of UI after treatment. Among all patients, 55.4% reported very good QoL. These reports of good QoL by patients could be due to their very good adjustment to the situation, with regard to being diagnosed with and receiving treatment for cancer, or due to the patients considering UI to not be much of an issue. Additionally, 34% of patients reported they had not been informed about the risk of UI before treatment. Informing patients about UI as one of the risks of therapy before initiating the treatment is crucial as patients who had been informed beforehand coped with UI far better than those who were not informed. Hence, the treatment of UI is often successful, so patients should be encouraged to receive urogynecological consultation.

**Supplementary Information:**

The online version contains supplementary material available at 10.1186/s12905-022-01999-1.

## Introduction

UI is uncontrolled and involuntary urine leakage and is a prevalent condition found within society. In addition to psychological and physical suffering for those affected, UI often leads to stigmatization and, in most cases, remains undertreated. Depending on the sources, UI has a prevalence between 5 and 50% in Germany, which is higher among women [1].

The many risk factors for UI include menopause, vaginal atrophy, hormone replacement therapy, genitourinary surgery (for example, hysterectomy), RTx, stroke, depression, dementia, diabetes, and obesity [2–8]. Both the prevalence and severity of UI increase with age [9]. Older age and severe symptoms are factors most strongly associated with seeking help [10, 11]. Wu et al. demonstrated a bimodal peak for the age of developing stress urinary incontinence (SUI), initially at age 46 and then again at ages 70–71, with an associated annual risk of SUI development at 3.8 and 3.9 per 100,000 women, respectively [12].

The probability of developing malignant tumors also increases with age, especially in the cases of malignancies that occur in the abdominal cavity and pelvic regions. Due to the current therapeutic options for malignancies in the pelvis, such as RTx, CTx, surgery, and ABT, the survival rates have significantly improved [13]. In many cases, malignancy can be viewed as a chronic disease.

Gyneco-oncological surgeries include total hysterectomy, radical hysterectomy with or without diagnostics or therapeutic lymphadenectomy, adenectomy, and vulvectomy, which could be performed through diverse surgical access routes (for example, abdominal, vaginal, and laparoscopic routes). Important to note is that all of these treatments may directly or indirectly cause injury to the organs or structures of the pelvis, possibly affecting the physiology of the organs and limiting functionality. Since the urinary and genital tracts are closely interconnected, the risk for injury due to gyneco-oncological surgeries and RTx is very high. However, advances in gynecological surgical methods and the growing experience of gynecologists are steadily reducing this risk [14].

Complaints following treatment for gynecologic cancer may present as chronic pelvic pain or bladder voiding dysfunction, such as UI, bowel voiding dysfunction, lymphedema or sexual dysfunction, or intimate sensory dysfunction, all of which may affect patients' QoL. Thus, ultimately, with improvements in cancer survival, morbidity due to therapy and its impacts on QoL must also be considered to evaluate the treatment modality [15]. As also noted by Pizzol et al. [16], UI is almost certainly associated with poor QoL.

This study assessed, as primary objectives, the UI rate among the gyneco-oncological survivors at the University Hospital Aachen from January 2015 to August 2018 and its impact on the QoL of these women. Moreover, we analyzed the correlation between the therapeutic factors (type of therapy, surgical access route of the oncological therapy used, and the radicality of the OP) and UI. We also evaluated whether any correlation can be found between type of malignancy and UI.

The secondary aim of this study was to analyze the correlations between tumor type and the abovementioned therapeutic factors and QoL; furthermore, we aimed to address whether patients were suitably informed about the risk of UI before commencing these therapies.

## Materials and methods

Various methods and diagnostic tools, such as questionnaires, clinical examinations, and urodynamic studies with bladder-pressure measurement, are used to diagnose UI. The use of questionnaires allows for communication with standardized terminology. For example, the International Consultation on Incontinence Questionnaire-Short Form (ICIQ-UI SF) is a suitable questionnaire for recording symptoms; this questionnaire serves as a tool for both men and women to determine UI (frequency and severity) as well as its impact on their QoL and was thus used in this study. The ICIQ-UI SF contains four specific categories of questions: frequency of UI, amount of leakage, the overall impact of UI on QoL, and a self-diagnostic item. This questionnaire is also used worldwide in clinics and practices for rapid comprehensive detection of UI. The validation grade of ICIQ-UI SF, according to the international continence society recommendation, is grade A and is rated on a scale from 0 to 21 [17].

This study was a cross-sectional questionnaire-based study. A list of patients who received gyneco-oncological treatment for pelvic malignancies from January 2015 to August 2018 was compiled by the Cancer Registry Center of the University Hospital of Aachen. The inclusion criteria included diagnosis with gynecologic cancers who had gyneco-oncological treatments for a pelvic malignancy at the department of gyneco-oncology at the University Hospital Aachen from January 2015 to August 2018, patient consent, and an age older than 18 years. The exclusion criteria included refusal to participate, pregnancy, an age younger than 18 years, death at the time of enrolment, not undergoing any gyneco-oncological treatment at the University Hospital of Aachen between January 2015 and August 2018, and the presence of a neobladder.

The patients were mailed informational, privacy, and informed consent forms in advance. These forms provided information about the study as well as the questionnaires. After a three-month period of patient inclusion into the study, some patients’ records were returned with their responses. Additionally, some documents were returned without responses due to undeliverability.

A second attempt was made to contact these patients by telephone, assuming that their postal addresses were incorrect, and the patients who did not initially respond were again asked for consent to participate in the study.

If consent was obtained, the information forms, privacy statements, consent forms, and questionaries were again sent to the patients. Once the consent forms were signed, the patients were contacted by telephone and the questionnaires were completed via phone call. All questions were asked neutrally, and all answers were gathered in an unbiased and objective manner. During this telephone interview, patients were asked whether they experienced de novo UI or worsening of pre-existing UI after their therapy. Data about UI were collected using the validated questionnaire ICIQ-UI SF.

The patients who had UI were also asked if UI affected their life. Their answers should be considered subjective and ranged from 0 (not affected by UI) to 10 (extremely affected by UI). Moreover, they were asked about their QoL; QoL was assessed using a scale ranging from 0 (severely affected by UI and with very poor QoL) to 7 (very good QoL).

The patients were also asked whether they had been informed about the risk of UI as a possible complication of their oncological treatment.

The patients who were enrolled were diagnosed with cervical CA, ovarian CA, vulvar CA, endometrial CA, peritoneal CA, vaginal CA, immature ovarian teratoma, ovarian granulosa cell tumor, malignant mixed Müllerian tumor (MMMT), fallopian tube cancer, uterine sarcoma, and dysgerminoma.

**Fig. 1:**
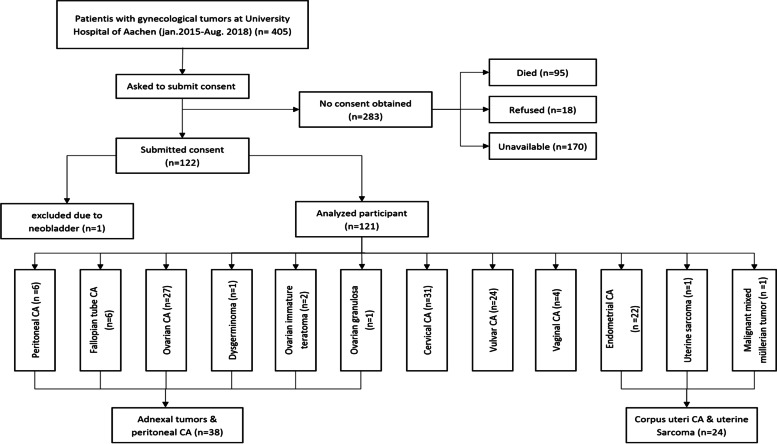
Study enrollment and tumor classification

Due to the small number of patients with some of these types of malignancies, all types of gynecological CAs were categorized into the following groups: cervical CA; adnexal tumor and peritoneal cancer (AT/Perit. CA), which included ovarian CA, peritoneal CA, fallopian tube CA, immature ovarian teratoma, ovarian granulosa cell tumor, and dysgerminoma; vulvar CA; corpus uteri CA and uterine sarcoma, which included endometrial CA, MMMT, and uterine sarcoma; and vaginal CA (Fig. 1). For the multivariate analysis, the vaginal CA and vulvar CA groups were combined.

### Statistical analysis

All variables were summarized as descriptive statistics. The continuous variables are expressed as mean values ± standard deviation (SD). The categorical data are presented as absolute frequencies and percentages. Additionally, boxplots were used to compare the ICIQ values for different tumor types, surgical treatment (Yes/No), and other subgroups.

The ICIQ and QoL scores were modelled by different multivariate regression models to identify possible predictors for UI and to investigate their influence. For all models, only the best-fitting models based on a sophisticaed model selection algorithm are reported in the Results section.

All tests were two-sided and assessed at the 5% significance level. Because of the exploratory nature of the study, the significance level was not adjusted to account for multiplicity. All statistical analyses were conducted using the statistical software R [18]. For a detailed description of the statistical analyses, we refer the reader to the Additional file 1.

## Results

Four hundred and five women were treated at the University Hospital of Aachen for pelvic malignancies from January 2015 to August 2018. A total of 95 patients died during this period of time. Eighty-seven patients could not be reached by telephone. One patient had a neobladder. The telephone numbers of 83 patients were incorrect, and 18 patients refused to participate in the study. Therefore, a total of 284 patients were excluded and 121 patients were included.

The age range was from 29 to 90 years, and the mean age was 62.3 years. The BMI ranged from 16 to 63.2, the mean of which was 27.3.

Eighty-eight patients (72.7%) had a minimum of one childbirth, and seventeen of them (14%) had a minimum of one c-section.

The distribution of tumor types among the 121 women with malignancy was as in Table 1.

**Table 1:** Distribution of malignancies among patients before and after categorization

Type of malignancy	Number of patients	Tumor group after categorization	Number of patients
Ovarian CA	27 (22.3%)	AT/Perit. CA	38 (31.4%)
Peritoneal CA	6 (5%)		
Immature ovarian teratoma	2 (1.7%)		
Ovarian granulosa cell tumor	1 (0.8%)		
Fallopian tube CA	1 (0.8%)		
Dysgerminoma	1 (0.8%)		
Endometrial CA	22 (18.2%)	CU/US	24 (19.8%)
MMMT	1 (0.8%)		
Uterine sarcoma	1 (0.8%)		
Vulvar	24 (19.8%)	Vulvar	24 (19.8%)
Vaginal	4 (3.3%)	Vaginal	4 (3.3%)
Cervical	31 (25.6%)	Cervical	31 (25.6%)

In total, 59 patients (48.8%) reported having no UI, 16 women (13.2%) already suffered from UI before their oncological therapy and reported an exacerbation due to the treatment, and 46 (38%) patients had de novo UI.

The prevalence of each category of ICIQ scores among the patients is shown in Table 2.

**Table 2:** Prevalence of each category of ICIQ scores

ICIQ score	0	1–5	6–12	13–18	19–21
Severity of UI	–	(Slight)	(Moderate)	(Severe)	(Very severe)
Number of patients	59 (48.8%)	18 (14.9%)	31 (25.6%)	11 (9.1%)	2 (1.7%)

In this study, 116 patients (95.9%) had undergone gynecological surgeries: in 52 patients (43%), the OP was performed laparoscopically, 57 patients (47.1%) had laparotomies; 31 patients (25.6%) had vaginal or vulvar surgeries; 84 women (69.4%) underwent hysterectomies; 22 patients (18.2%) had radical hysterectomies; 56 patients (46.3%) had pelvic and para-aortic lymphadenectomy; and CTx, RTx, and ABT were performed in 51 patients (42.2%), 31 patients (25.6%), and 11 patients (9.1%), respectively.

The rate of malignancy recurrence was also recorded: 22 patients (78.6%) had one recurrence, 3 patients (2.5%) had two recurrences, 1 patient (0.8%) had three recurrences, 1 patient (0.8%) had four recurrences, and 1 patient (0.8%) had seven recurrences.

Regarding the preoperative information, 57 women (47.1%) reported that they were well informed about the potential risk of preoperative UI as one of the possible complications of their oncological therapy.

Additionally, 22 patients (18.2%) were unsure whether they had been informed about this risk and 42 patients (34.7%) confirmed that they had not been notified preoperatively.

Among the patients with UI, 15 (24.2%) of them reported that UI did not affect their life.

This study shows that 67 women (55.4%) have a very good QoL. Additionally, we show that 25 (40.3%) patients with UI have a QoL score ≥ 5.

Table 3 shows that the severity of the impact that UI had on patients' lives.

**Table 3:** Distribution of patients whose lives were affected by UI

Score	1–3	4–6	7–9	10
Severity	(Slight)	(Moderate)	(Severe)	(Very severe)
Number of patients	19	18	8	2

The distribution of reported QoL scores among the patients is shown in Table 4.

**Table 4:** Prevalence of QoL score, ranging from 0 (extremely poor QoL) to 7 (very good QoL)

QoL score	0 (very poor)	1–2	3–4	5–6	7 (very good)
Number of patients	3 (2.5%)	8 (6.6%)	26 (21.5%)	17 (14.1%)	67 (55.4%)

**Fig. 2:**
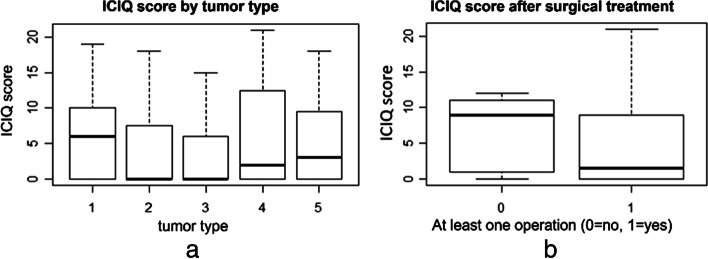
**a** Bivariate analysis between ICIQ score and tumor category (1: cervical cancer, 2: CU/US, 3: AT/Perit. CA, 4: vaginal cancer, and 5: vulvar cancer). **b** Bivariate analysis between ICIQ score and surgical treatment

Fifty percent of our collective had an ICIQ score between 3 and 21, with a median of 3. The bivariate analysis demonstrated that the median ICIQ score in the cervical cancer group, with a score of 6, was higher than this score in other groups of tumor types (Fig. 2a). Moreover, the bivariate analysis between ICIQ score and operative therapy showed that the women who underwent at least one surgery in their pelvic cavity or pelvic floor had lower median ICIQ scores compared with those who did not have any surgeries (1.5 vs. 9). However, the ICIQ score in 25% of patients in the surgery subgroup was between 9 and 21, and the sample size in the no-surgery subgroup was small (five women) (Fig. 2b).

**Fig. 3:**
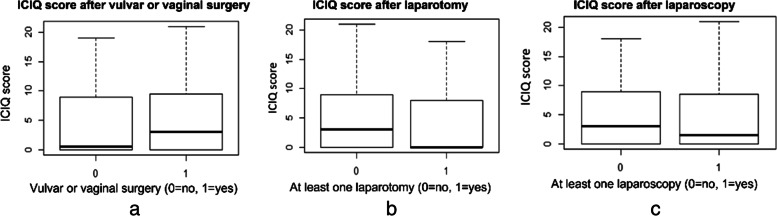
**a** Bivariate analysis between ICIQ score, and vulvar or vaginal surgery. **b** Bivariate analysis between ICIQ score and laparotomy. **c** Bivariate analyses between ICIQ score and laparoscopy

In addition, the bivariate analysis revealed that the patients who had undergone vulvar or vaginal surgeries had higher median ICIQ scores compared with those who did not have this type of surgical access route (3 vs. 0.5) and that they had even higher median ICIQ scores than those who underwent laparotomy or laparoscopy (3 vs. 0 and 3 vs. 1.5, respectively) (Fig. 3 a, b, c).

**Fig. 4:**
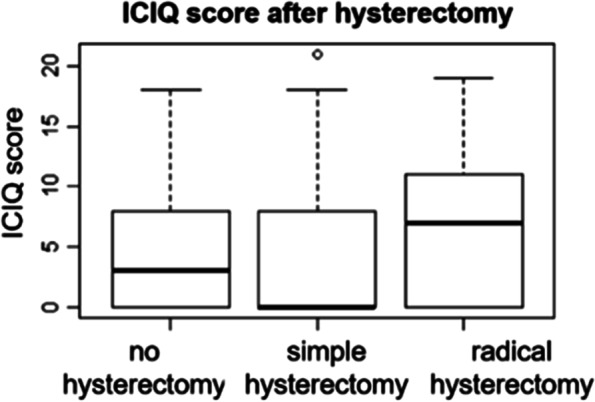
Bivariate analysis between ICIQ score and hysterectomy radicality

The intensity of UI was also observed in women who received radical hysterectomy or simple hysterectomy, or even in those who did not undergo either. The study showed higher median ICIQ scores in patients with radical hysterectomy than in those who had undergone simple hysterectomy and in those who did not undergo either (median ICIQ scores of 7, 0, and 3, respectively) (Fig. 4).

**Fig. 5:**
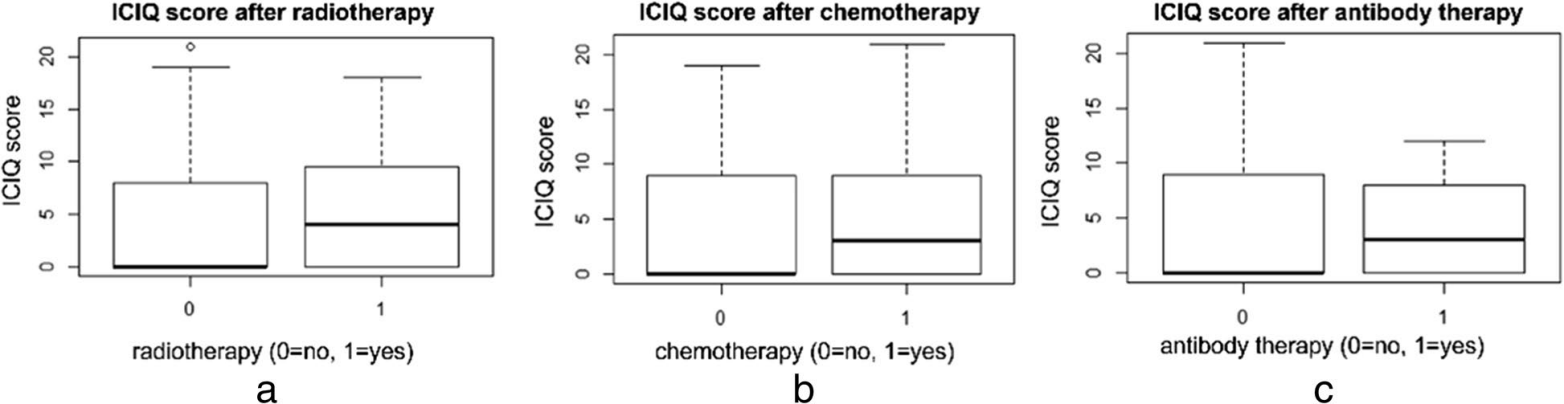
**a** Bivariate analysis between ICIQ score and RTx. **b** Bivariate analysis between ICIQ score and CTx. **c** Bivariate analysis between ICIQ score and ABT

ICIQ score was also evaluated in patients who received RTx, CTx, or ABT. In all of these groups, the median ICIQ scores (4, 3, and 3, respectively) were higher than in those who did not undergo these therapies (median ICIQ score of 0) (Fig. 5 a, b, c).

A subgroup analysis of ICIQ scores by tumor and therapy types revealed the median ICIQ scores illustrated in Table 5.

**Table 5:** Subgroup bivariate analysis among ICIQ scores, tumor groups, and therapy types

Therapy types	No OP	OP	No RTx	RTx	No CTx	CTx	No ABT	ABT
**Tumor groups**								
**Cervical CA**	10	4	3	7	6	5.5	5	7
**CU/US**	0	0	0	0	0	8	0	N/A
**AT/Perit. CA**	1	0	0	N/A	0	0.5	0	3
**Vulvar**	12	3	0	9	3	9	2	N/A
**Vaginal**	N/A	2	2	N/A	0	21	3	0

This study found no significant association between UI intensity and tumor types. In addition, no significant correlation was found among ICIQ score and age, childbirth, and c-section.

According to the best model, the multivariate analysis incorporating all variables as possible predictors revealed that BMI (*p* = 0.05), radical hysterectomy (*p* = 0.02), vulvar or vaginal surgery (*p* = 0.05), and presence of UI before treatment (*p* = 0.004) have significant impacts on the presence of UI (ICIQ score > 0). They decreased the probability of having an ICIQ score of 0. This statistical model also showed a slight association between malignancy recurrences (*p* = 0.07) and an ICIQ score of 0. The higher the rate of malignancy recurrences, the lower the probability of having an ICIQ score of 0 (Table 6).

**Table 6:** Estimate and *p*-value of variables as possible predictors for the presence of UI in multivariate analysis (zero-inflated model)

Variables	Estimate	*p*-value
BMI	− 0.06	0.05
Radical hysterectomy	− 1.91	0.02
Vulvar or vaginal surgery	− 1.25	0.05
Presence of UI before treatment	− 4.33	0.004
Malignancy recurrences	− 0.54	0.07

The best model also assessed which factors played roles in increasing the ICIQ score in cases with UI (ICIQ score > 0), and demonstrated that ABT (*p* = 0.001), laparoscopic surgery (*p* = 0.01) and laparotomy (*p* = 0.03) have negative effects on increasing of the ICIQ score in cases with UI (ICIQ score > 0). However, simple hysterectomy (*p* =  < 0.001), radical hysterectomy (*p* =  < 0.001), vulvar or vaginal surgical route access (*p* = 0.02), and CTx (*p* = 0.03) have positive effects on increasing the ICIQ score and thus worsening UI in cases with UI (ICIQ > 0) (Table 7).

**Table 7:** Estimate and *p*-value of variables as possible predictors for increasing ICIQ score in cases with UI in multivariate analysis (zero-inflated model)

Variables	Estimate	*p*-value
ABT	− 0.46	0.001
Laparoscopic surgery	− 0.37	0.01
Laparotomy	− 0.34	0.03
Simple hysterectomy	0.72	< 0.001
Radical hysterectomy	0.70	< 0.001
Vulvar or vaginal surgical route access	0.26	0.02
CTx	0.25	0.03

According to the best model, the multivariate analysis demonstrated no association between tumor types and hysterectomy with QoL but showed that the following factors decrease the probability of having a very good QoL (QoL score of 7) in cases with pelvis malignancies: high BMI (*p* = 0.09), high rate of malignancy recurrences (*p* = 0.04), vulvar or vaginal operation (*p* = 0.03), pelvic and para-aortic lymphadenectomy (*p* = 0.18), the presence of UI before the treatment (*p* = 0.01), and the time between the first diagnosis and the collection of questionnaire responses (*p* = 0.18). However, both factors, operation and having no c-section, increased the probability of having a very good QoL (Table 8).

**Table 8:** Estimate and *p*-value of variables that have an impact on the probability of having a very good QoL in multivariate analysis (zero-inflated model)

Variables	Estimate	*p*-value
BMI	− 0.06	0.09
Rate of malignancy recurrences	− 0.80	0.04
Vulvar or vaginal operation	− 1.30	0.03
Pelvic and para-aortic lymphadenectomy	− 0.77	0.18
The presence of UI before the treatment	− 3.07	0.01
No c-section	0.41	0.02
Operation	4.49	0.01
The time between the first diagnosis	− 0.02	0.18

The QoL of patients with pre-existing UI prior to their gynecological cancer being diagnosed and treated improved after the therapy but was not statistically significant (*p* = 0.16). This study also showed that high BMI (*p* = 0.17), vulvar or vaginal surgery (*p* = 0.002), and pelvic and para-aortic lymphonodectomy (p = 0.14) worsened the QoL for those with QoL impairments (Table 9).

**Table 9:** Estimate and *p*-value of variables that have an impact on worsening or improving QoL multivariate analysis (zero-inflated model)

Variables	Estimate	*p*-value
BMI	0.01	0.17
Vulvar or vaginal surgical route access	0.54	0.002
Pelvic and para-aortic lymphadenectomy	0.27	0.14
Presence of UI before treatment	− 0.30	0.16

The Spearman correlation analysis revealed a strong correlation between the ICIQ and QoL scores and demonstrated that the patients with low ICIQ scores tend to have higher QoL scores and vice versa (correlation coefficient of − 0.80).

## Discussion

The risks for both malignancy and UI increase with increasing age. As previously mentioned, due to improvements in diagnostic methods and therapeutic advancements, many CAs can be considered chronic diseases; therefore, the QoL of patients with malignancy becomes even more important.

UI is a condition that can affect QoL, and in general, the treatment of UI is highly successful in most cases and leads to particularly good results. In some cases, the treatment of UI can be achieved with lifestyle modifications such as weight loss, and physical therapy, biofeedback therapy, medication, or surgery.

Our study was conducted to assess whether the risk of UI increases with certain gynecological pelvic malignancies or specific gyneco-oncological therapies and to identify any associations between these malignancies or therapies, and the ICIQ score, which shows the presence and the severity of UI. The study also aimed to identify if UI in the presence of malignancy influences the QoL of affected patients.

Except for a study by Alicja Ziętek-Strobl et al. [19], no study has investigated the relationship between the occurrence of UI or its worsening after gyneco-oncological treatments, and the type of gyneco-oncological tumor. They studied the urogynecological symptoms among 160 oncological survivors and the impact of oncological therapy on pelvic floor disorders and lower urinary tract symptoms during a 6-month follow-up period.

Out of the 405 patients contacted by mail in our study, we initially received responses from only 50 patients out of the 355 patients who were alive. After conducting telephone calls and interviews with all patients, we included an additional 72 patients in the study. Some of these 72 patients reported that they were not aware of their UI, and they thought they would have to undergo further examinations as part of the study, so they did not respond to the mail. Some women were still uncomfortable talking about their UI, considering that UI is still a taboo subject for many women. Some cases did not expect UI as a result of their malignancy or as a complication of their oncological therapy. Some patients reported that UI is not important compared with their main diagnosis and treatment.

Additionally, 18 patients were not willing to have any sort of unnecessary contact with the hospital because it reminded them of the difficult time they experienced there, so they refused to participate in the study although the questionnaire could have been answered in less than 5 min. The research by Yngvild S. Hannestad et al. showed that, of 6625 women with UI who were 20 years or above, only one-quarter of those with any incontinence and half of those with significant incontinence had consulted a physician [11].

In our study, 59 patients (48.8%) reported no UI, although most had many known risk factors for UI; 46 patients (38%) reported having de novo UI; and 16 (13.2%) reported worsening incontinence symptoms since their gyneco-oncological treatments. Alicja Ziętek-Strobl et al. [19] showed a lower incidence of de novo UI (25 patients, 15.6%) and reported UI in 48.7% of their patients. In a cross‐sectional study, Erekson et al. [20] demonstrated that 52% of endometrial cancer survivors suffered from at least moderate UI.

Our investigation found no association between ICIQ score and tumor types, similar to the study by Alicja Ziętek-Strobl et al. [19].

Lukacz et al. [21] showed that childbearing leads to an increase in UI; Noriko Nakayama et al. [22] observed multiple c-sections as a risk factor for UI after surgical treatment of gynecologic CA. However, both childbearing and c-section could not be shown as factors that impacted the ICIQ score in our study.

In addition, no significant correlation was found between the ICIQ score and age in our research. In contrast, Hannestad YS et al. reported that UI’s prevalence and severity increase with age [9]. This difference might be due to the small sample size of our study.

BMI, radical hysterectomy, vulvar or vaginal surgical access routes, and the presence of UI before initiation of the therapy are factors shown to have significant impacts on the presence of UI after the gyneco-oncological treatment in our study (ICIQ score > 0). These factors significantly decrease the probability of having an ICIQ score of 0 after therapy. A study by the U.S. National Library of Medicine found that 30% of all cases of UI are patients who have undergone gynecologic surgeries. Among these cases, abdominal hysterectomy and reconstructive vaginal surgery are the most important gynecologic surgeries associated with UI [23]. Ceccaroni et al. [24] showed UI rates of 55% after radical hysterectomy.

Additionally, we identified that laparoscopy and laparotomy could significantly decrease UI intensity among the gyneco-oncological survivors after their treatment. This result may be due to the advances in gynecological surgical methods and the growing experience of gynecologists. Additionally, ABT was revealed as a factor that could reduce UI intensity. Conducting studies with large sample sizes is suggested for better evaluations of the impact of this factor on UI.

Our analysis also indicated a strong negative correlation between the ICIQ and QoL scores and showed that the patients with low ICIQ score tended to have higher QoL score. As also noted by Pizzol et al. [16], UI is almost certainly associated with poor QoL. The research by Coyne, K. S. et al. has also highlighted that the development of UI as a complication of gyneco-oncological treatment was associated with anxiety and depression [25]. However, in our work, 25 patients (40.3%) who suffered from UI reported having good and very good QoLs.

We also found that the patients with a presence of UI prior to treatment could have significantly better QoL after their treatment. This result could be due to their very good adjustment to the situation, with regard to being diagnosed with and receiving treatment for cancer, or due to the patients considering UI to not be much of an issue in comparison with dealing with CA, which is a shocking and life-changing issue for them and may have made the UI seem less important to them.

About one third of patients reported that they had not been informed of the risk of UI before the initiation of treatment; however, this percentage could be partly due to recall bias. Prospective studies should be conducted to minimize recall bias.

Assessments of patients regarding neurological-related diseases such as dementia, transient ischemic attack (TIA), Alzheimer’s disease, and cerebrovascular accident (CVA) as possible causes of UI are suggested as future studies.

## Conclusions

Our study reported having de novo UI in approximately 38% of gyneco-oncological survivors. Additionally, we showed the worsening of incontinence symptoms in 13.2% of patients since their treatments.

We found no significant association between the ICIQ score, indicating the presence and intensity of UI and the tumor types. In addition, no significant correlation was found between ICIQ score and age, childbearing, and c-section.

BMI, radical hysterectomy, vulvar or vaginal surgical access routes, and the presence of UI before the initiation of therapy were found to be factors that significantly decreased the probability of having an ICIQ score of 0 after the gyneco-oncological treatment. In contrast, we identified that ABT, laparoscopy, and laparotomy could significantly decrease the intensity of UI among the gyneco-oncological survivors after their treatment. Therefore, we could provide patients with more information about the risks of UI following their treatment.

Our analysis also showed that patients with lower UI intensity tended to have higher QoL scores than patients with severe UI.

We could not find any association between tumor types and QoL; however, the study showed that high BMI, a high rate of malignancy recurrences, vulvar or vaginal operations, and the presence of UI before treatment decreased the probability of having a very good QoL (QoL score of 7) in cases with pelvis malignancies. Additionally, our study indicated a slight association between high BMI and worsening of the QoL for those with QoL impairments. Therefore, a normal BMI could positively be associated with having a better QoL.

According to telephone interviews, women who were informed about the risk of UI before their therapy expressed that they were able to cope better with UI. However, the patients who reported that they had no information about the risk of UI after their therapy and its possible treatment options had not yet discussed their micturition problems with their doctors.

Therefore, it is particularly important for women to be well informed about the risk of de novo UI or the deterioration of UI as a possible complication before starting therapy to improve acceptance of this potential risk. In addition, these women should be notified that UI can affect their QoL and that, in the case of UI, they can seek urogynecological consultation for diagnosis and possible treatment to improve their QoL.

In our study, during the phone calls, the patients with existing bladder voiding disorders were given detailed advice, and if necessary, a presentation for further clarification and treatment of the complaints was organized in our urogynecological department.

## Supplementary Information

**Additional file 1.** Detailed description of the statistical analyses.

## Data Availability

The datasets used and/or analyzed during the current study are available from the corresponding author upon reasonable request.

## Abbreviations

ABT: Antibody therapy
AIC: Akaike information criterion
AT/Perit. CA: Adnexal tumor and peritoneal cancer
BMI: Body mass index
CA: Cancer
c-section: Cesarean section
CTx: Chemotherapy
CU/US: Corpus uteri cancer and uterine sarcoma
CVA: Cerebrovascular accident
ICIQ score: International consultation on incontinence questionnaire score
ICIQ-UI SF: Incontinence questionnaire-short form
MMMT: Malignant mixed müllerian tumor
N/A: Not applicable
OP: Operation
QoL: Quality of life
RTx: Radiotherapy
SD: Standard deviation
SUI: Stress urinary incontinence
TIA: Transient ischemic attack
UI: Urinary incontinence

## References

[1] Niederstadt C, Gaber E, Füsgen I. Harninkontinenz. Berlin: Robert Koch-Inst; 2007. p. 47. (Gesundheitsberichterstattung des Bundes).

[2] Lawrence JM, Lukacz ES, Liu ILA, Nager CW, Luber KM (2007). Pelvic floor disorders, diabetes, and obesity in women. Diabetes Care.

[3] Jackson SL, Scholes D, Boyko EJ, Abraham L, Fihn SD (2005). Urinary incontinence and diabetes in postmenopausal women. Diabetes Care.

[4] Phelan S, Grodstein F, Brown JS (2009). Clinical research in diabetes and urinary incontinence: what we know and need to know. J Urol.

[5] Melville JL (2005). Urinary incontinence in us women: a population-based study. Arch Intern Med.

[6] Brown JS, Sawaya G, Thom DH, Grady D (2000). Hysterectomy and urinary incontinence: a systematic review. The Lancet.

[7] Drennan VM, Rait G, Cole L, Grant R, Iliffe S (2013). The prevalence of incontinence in people with cognitive impairment or dementia living at home: a systematic review: prevalence of incontinence in people with dementia at home. Neurourol Urodyn.

[8] Subak LL, Richter HE, Hunskaar S (2009). Obesity and urinary incontinence: epidemiology and clinical research update. J Urol.

[9] Hannestad YS, Rortveit G, Sandvik H, Hunskaar S (2000). A community-based epidemiological survey of female urinary incontinence. J Clin Epidemiol.

[10] Harris SS, Link CL, Tennstedt SL, Kusek JW, McKinlay JB (2007). Care seeking and treatment for urinary incontinence in a diverse population. J Urol.

[11] Hannestad Y, Rortveit G, Hunskaar S (2002). Help-seeking and associated factors in female urinary incontinence. The Norwegian EPINCONT study. Scand J Prim Health Care.

[12] Wu JM, Matthews CA, Conover MM, Pate V, Funk MJ (2014). Lifetime risk of stress incontinence or pelvic organ prolapse surgery. Obstet Gynecol.

[13] Suh DH, Kim JW, Kang S, Kim HJ, Lee KH (2014). Major clinical research advances in gynecologic cancer in 2013. J Gynecol Oncol.

[14] Gilmour DT, Dwyer PL, Carey MP (1999). Lower urinary tract injury during gynecologic surgery and its detection by intraoperative cystoscopy. Obstet Gynecol.

[15] Ramaseshan AS, Felton J, Roque D, Rao G, Shipper AG, Sanses TVD (2018). Pelvic floor disorders in women with gynecologic malignancies: a systematic review. Int Urogynecology J.

[16] Pizzol D, Demurtas J, Celotto S, Maggi S, Smith L, Angiolelli G (2021). Urinary incontinence and quality of life: a systematic review and meta-analysis. Aging Clin Exp Res.

[17] ICIQ-UI SF. ICIQ. [cited 2022 Mar 15]. Available from: https://iciq.net/iciq-ui-sf.

[18] R: The R Project for Statistical Computing. [cited 2022 Apr 5]. Available from: https://www.r-project.org/.

[19] Ziętek-Strobl A, Futyma K, Kuna-Broniowska I, Wojtaś M, Rechberger T (2020). Urogynaecological symptoms among oncological survivors and impact of oncological treatment on pelvic floor disorders and lower urinary tract symptoms. A six-month follow-up study. J Clin Med.

[20] Erekson EA, Sung VW, DiSilvestro PA, Myers DL (2009). Urinary symptoms and impact on quality of life in women after treatment for endometrial cancer. Int Urogynecol J.

[21] Lukacz ES, Lawrence JM, Contreras R, Nager CW, Luber KM (2006). Parity, mode of delivery, and pelvic floor disorders. Obstet Gynecol.

[22] Nakayama N, Tsuji T, Aoyama M, Fujino T, Liu M (2020). Quality of life and the prevalence of urinary incontinence after surgical treatment for gynecologic cancer: a questionnaire survey. BMC Womens Health.

[23] Thüroff JW, Abrams P, Andersson KE, Artibani W, Chapple CR, Drake MJ (2011). EAU guidelines on urinary incontinence. Eur Urol.

[24] Ceccaroni M, Roviglione G, Spagnolo E, Casadio P, Clarizia R, Peiretti M (2012). Pelvic dysfunctions and quality of life after nerve-sparing radical hysterectomy: a multicenter comparative study. Anticancer Res.

[25] Coyne KS, Wein AJ, Tubaro A, Sexton CC, Thompson CL, Kopp ZS (2009). The burden of lower urinary tract symptoms: evaluating the effect of LUTS on health-related quality of life, anxiety and depression: EpiLUTS. BJU Int.

