# Supplementary material for: Evaluation of urinary continence status and its influence on quality of life after gyneco-oncological treatment of female pelvic malignancies at an oncological center
Source: BMC Womens Health. 2022 Oct 25;22:422. doi: 10.1186/s12905-022-01999-1 (PMC9594888; doi:10.1186/s12905-022-01999-1)
Supplement: Supplementary file 1 — Additional file 1. Detailed description of the statistical analyses. [file 12905_2022_1999_MOESM1_ESM.docx]

Detailed description of the statistical analysis:

All variables were summarized as descriptive statistics. The continuous variables are expressed as mean values ± standard deviation (SD). The categorical data are presented as absolute frequencies and percentages.

In the first bivariate descriptive analysis, comparison boxplots and five-point summaries (minimum, lower quartile, median, upper quartile, and maximum) for the International Consultation on Incontinence Questionnaire (ICIQ) scores were obtained for each predictive factor.

For the primary analysis, the ICIQ score was regarded as the primary endpoint. To identify possible predictors for UI and to investigate their influence, various statistical models were fitted to the ICIQ score. Linear regression was conducted first to apply an easy, well-known method that has several advantages regarding its interpretation. However, the ICIQ score is not measured on a continuous scale with countable data. Due to overdispersion and due to the high occurrence of zeros (59 patients reported ICIQ scores of zero, comprising 48.76 % of all observations), instead of fitting the Poisson or negative binomial models, a zero-inflated regression model was applied(1,2).

The zero-inflated model used a mixture specification with a logistic component that models the probability of an ICIQ score being equal to zero. For the counter-probability of the ICIQ score not being equal to zero, a Poisson count component was used to model the ICIQ score.

As a sensitivity analysis, logistic regression was performed on a dichotomized endpoint, taking a value of 1 if the ICIQ score was below or equal to 3 and a value of 0 if the ICIQ score was above 3.

For all models (including the sensitivity analysis), model selection was performed using the Akaike information criterion (AIC). The best-fitting model with the lowest AIC is reported in the Results section.

The above analyses for the ICIQ score were repeated for the QoL score after subtracting the score from 7 to obtain inflation at zero instead of 7 (55.37 % originally reported a QoL of 7).

All tests were two-sided and assessed at the 5% significance level. Because of the exploratory nature of the study, the significance level was not adjusted to account for multiplicity. All statistical analyses were conducted using the statistical software R (3)

While a Poisson (Quasi-Poisson due to overdispersion) or negative binomial model yielded an expected number of zero values of only 6.47 % or 41.0 %, respectively, the zero-inflated model yielded an expected number of 48.755 %, very close to the actual 48.760 % of zero values in the dataset. A superior model fit was also demonstrated by the mean absolute error of predictions. While the zero-inflated model without any variables (information) predicted ICIQ incorrectly by an expected margin of 4.85, the linear and negative binomial model obtained expected errors ranging from 4.03 to 4.14 while the zero-inflated model obtained expected errors ranging from 3.42 to 3.88.

References

1. Lambert D. Zero-Inflated Poisson Regression, with an Application to Defects in Manufacturing. Technometrics. 1992;34(1):1–14.

2. Kleiber C, Zeileis A. Applied econometrics with R. Berlin: Springer Science & Business Media; 2008. p. 229.

3. R: The R Project for Statistical Computing. [cited 2022 Apr 5]. Available from: https://www.r-project.org/
